# NMRFAM-SDF: a protein structure determination framework

**DOI:** 10.1007/s10858-015-9933-8

**Published:** 2015-04-22

**Authors:** Hesam Dashti, Woonghee Lee, Marco Tonelli, Claudia C. Cornilescu, Gabriel Cornilescu, Fariba M. Assadi-Porter, William M. Westler, Hamid R. Eghbalnia, John L. Markley

**Affiliations:** National Magnetic Resonance Facility at Madison, Biochemistry Department, University of Wisconsin-Madison, 433 Babcock Drive, Madison, WI USA

**Keywords:** ADAPT-NMR, ARECA, Automated protein structure determination framework, CASD-NMR, Non-uniform sampling, PINE, PONDEROSA-C/S, Validation

## Abstract

The computationally demanding nature of automated NMR structure determination necessitates a delicate balancing of factors that include the time complexity of data collection, the computational complexity of chemical shift assignments, and selection of proper optimization steps. During the past two decades the computational and algorithmic aspects of several discrete steps of the process have been addressed. Although no single comprehensive solution has emerged, the incorporation of a validation protocol has gained recognition as a necessary step for a robust automated approach. The need for validation becomes even more pronounced in cases of proteins with higher structural complexity, where potentially larger errors generated at each step can propagate and accumulate in the process of structure calculation, thereby significantly degrading the efficacy of any software framework. This paper introduces a complete framework for protein structure determination with NMR—from data acquisition to the structure determination. The aim is twofold: to simplify the structure determination process for non-NMR experts whenever feasible, while maintaining flexibility by providing a set of modules that validate each step, and to enable the assessment of error propagations. This framework, called NMRFAM-SDF (NMRFAM-Structure Determination Framework), and its various components are available for download from the NMRFAM website (http://nmrfam.wisc.edu/software.htm).

**Electronic supplementary material:**

The online version of this article (doi:10.1007/s10858-015-9933-8) contains supplementary material, which is available to authorized users.

## Introduction

NMR spectroscopy has emerged as the premier approach for obtaining information about biomolecular interactions, structural dynamics, and three-dimensional structure in solution. However, the collection, processing, interpretation, and validation of NMR data remain challenging, and present barriers to more widespread applications. Efforts in the NMR community in the past two decades have focused on the automation of discrete steps involved in analyzing NMR data. More specifically, streamlining the overall sequence of steps in the procedure of protein structure calculation has received considerable attention (Lopez-Mendez and Guntert 2006; Serrano et al. 2012). The goal of the CASD-NMR competitions has been to foster the development of automated methods that lead to structures whose quality approaches those determined by tedious manual methods (Rosato et al. 2009, 2012).

The common process for NMR protein structure calculation begins with collecting NMR data for a number of through-bond and through-space experiments that will be processed into the frequency domain representation. A peak identification step, called peak-picking, is required to identify the signals of interest in the processed data. The chemical shifts of the peaks are assigned to the atoms of the backbone and side chains, and the assigned chemical shifts are used as labels for identifying NOE cross peaks in the NOESY spectra. These cross peaks provide spatial restraints for the 3D structure of the protein in the study (Clore and Gronenborn 1987, 1991; Wüthrich 1986). Spatial restraints, along with an empirical force-field, are then used to arrive at an ensemble of low energy structures that satisfy most of the restraints.

Long data acquisition times are a potential limiting factor in NMR studies, particularly with unstable targets, and a number of approaches have been developed for improving data acquisition through computational or experimental means (Bahrami et al. 2012; Brutscher 2013; Frydman et al. 2004; Hoch et al. 2007; 2014; Hyberts et al. 2012; Kim and Szyperski 2003; Kupce and Freeman 2003a; Lee et al. 2013; Lescop et al. 2007, 2009; Maciejewski et al. 2006; Orekhov et al. 2003; Orekhov and Jaravine 2011; Schanda and Brutscher 2005; Szyperski et al. 2002). Toward accelerating the data acquisition and consequently improving the sensitivity of the spectra, modifications in pulse programs have been introduced (Brutscher 2013; Frydman 2006; Lescop et al. 2007). Irregular or non-uniform sampling (NUS) schemes represent an alternative approach to conventional data collection (Bahrami et al. 2012; Hoch et al. 2007, 2014; Hyberts et al. 2012; Kim and Szyperski 2003; Kupce and Freeman 2003b; Maciejewski et al. 2006; Mobli and Hoch 2008; Orekhov et al. 2003; Orekhov and Jaravine 2011). Ultimately, the gains in time or sensitivity introduced by computational processes must be validated to ensure the robustness of signal identification—or peak picking. And, despite developments in peak picking algorithms (Alipanahi et al. 2009; Cheng et al. 2013; Chylla et al. 1998; Shin and Lee 2008; Tikole et al. 2014), the ability to deconvolve peaks in split or overlapped peaks remains unsatisfactory. Some data collection methods have the potential to distinguish between noise and peaks by employing a peak identification algorithm (Bahrami et al. 2012; Hiller et al. 2005; Kim and Szyperski 2003). However, for robust automation, validating the output from individual steps, or the combined steps of spectral processing and peak picking, remains a necessity.

Eghbalnia et al. (2005) and Bahrami et al. (2009) demonstrated that the computational problem of assigning protein chemical shifts from through-bond NMR experiments is of the class mathematicians call “NP-hard” (Bovet and Plerlulgi 1994). This infers a limitation on purely deterministic algorithms for chemical shift assignment or validation. Instead, it was proposed that automated chemical shift assignment approaches rely on non-deterministic or probabilistic algorithms (Bahrami et al. 2009, 2012; Schmidt and Guntert 2012), where a probabilistic validation process becomes optimal. Alternatively, when the chemical shift assignment method uses a deterministic algorithm in its core decision-making process (Jung and Zweckstetter 2004; MacRaild and Norton 2014; Xu et al. 2006), validation can utilize an accept-reject criterion, an approach that is suitable only when spectral signals are nearly complete and unambiguous.

The practice of structure determination by NMR spectroscopy involves a number discrete decision making steps that give rise to a non-linear relation between the inputs and outputs. The cumulative impact of nonlinear input–output relations could lead to unexpected and unpredictable errors. Stepwise and continuous validation can inform users of potential inconsistencies early in the process and flag them for optional correction; including manual corrections by users. Among existing data acquisition methods, ADAPT-NMR (Bahrami et al. 2012) provides a supporting verification GUI (graphical user interface), named ADAPT-NMR Enhancer (Lee et al. 2012). Other methods such as the ist@HMS (Hyberts et al. 2012) are designed with the goal of improving the sensitivity and resolution of multidimensional experiments by using non-uniform sampling data collection. More recently, the NESTA program (Sun et al. 2015) was developed to speed up the reconstruction of non-uniform sampled spectra thus making it more feasible for this method to be incorporated into high-throughput and automated approaches.

Accurate chemical shift assignment plays an important role in structure determination (Jee and Guntert 2003). The PINE (Probabilistic Interaction Network of Evidence) algorithm provides a probabilistically ranked set of possible assignments for every atom that users can use to investigate different possible candidates (Bahrami et al. 2009). The computational complexity of the chemical shift assignment for large proteins motivated us to introduce the PINE-SPARKY (Lee et al. 2009) to help users explore the possible assignments and validate the assignments by visualization on designated spectra. In addition to these probabilistic methods, a second category of assignment validation methods relies on chemical shift statistics (Moseley et al. 2004; Wang et al. 2005, 2010). Although useful, methods in this category do not consider the specific characteristics of the protein under study and therefore may cause false-negative and false-positive results (Dashti et al. 2015). This limitation is addressed by our recent introduction of ARECA, a probabilistic validation method that uses the NOESY spectra (or the corresponding peak lists) of the protein to validate the chemical shift assignments. The assessment of the reliability of chemical shift assignment (ARECA) package (Dashti et al. 2015) is the first probabilistic method that uses the large body of through-space statistics to validate chemical shift assignments. The CASD-NMR (Rosato et al. 2009, 2012) provided data-sets with raw and refined peaks that were used for evaluating ARECA in determining whether the assignments provided were consistent with the given NOESY peak lists.

The difficulty of the resonance assignment problem can increase when through-space (NOESY) experiments are considered—in this case, the number of peaks depends on the protein structure as well as the length of the sequence. A significant part of automation literature in NMR is focused on through-bond experiments (Bahrami et al. 2009; Hiller et al. 2005; Jung and Zweckstetter 2004; MacRaild and Norton 2014; Wu et al. 2006; Xu et al. 2006; Zimmerman et al. 1997) or mapping through-bond assignments into short-range NOESY contacts and predicting long-range NOE assignments (Güntert 2004; Herrmann et al. 2002; Lee et al. 2011, 2014a). This is, in part, a reflection of the additional computational complexity of NOE cross peak assignments (Linge et al. 2003; Schmidt and Guntert 2012), which includes the additionally complex task of extracting the distance restraints between the atoms. The ambiguities in assignment of long-range NOE cross peaks result in a set of intricate distance restraints that include a combination of ones that are correct and incorrect. Therefore finding the most suitable set of restraints to achieve an energetically favorable structure becomes a challenging optimization problem. The search for an optimal restraint set is usually performed by validation of the calculated intermediate structures and examination of the restraints used or discarded during the structure determination process (Güntert 2004; Herrmann et al. 2002; Kuszewski et al. 2004, 2008; Linge et al. 2003; Schwieters et al. 2003). The need for expertise in multiple areas (such as spectroscopic, structural, biochemical, and biophysical fields) and familiarity with several software tools makes this one of the most challenging remaining steps in NMR structure determination. PONDEROSA (Peak-picking Of NOE Data Enabled by Restriction of Shift Assignments) (Lee et al. 2011) addresses this challenge by automatically selecting peaks in the NOESY spectra and simultaneously interfacing with TALOS + (Shen et al. 2009), STRIDE (Frishman and Argos 1995) and CYANA (Güntert 2004) in an iterative process in order to identify the most reliable set of restraints. The recent introduction of PONDEROSA-C/S (Lee et al. 2014a) adds new functionality for user convenience by providing Ponderosa Client and Ponderosa Analyzer programs as interfaces to the core computational server (Ponderosa Server). In the course of developing PONDEROSA-C/S, data sets from CASD-NMR (Rosato et al. 2009, 2012) were used to evaluate and refine the algorithms in the Ponderosa Server. Ponderosa Analyzer is a reliable validation package for both identifying restraint violations and providing tools for investigating the structure and adjusting it to better fit to the experimental data. The package provides tools for visualizing the automatically generated restraints on the 3D structure and spectra by interfacing with PyMOL (DeLano and Lam 2005) and NMRFAM-SPARKY (Lee et al. 2014b). Other methods for structure validation include those that use statistics from structures in databases (Chen et al. 2010; Davis et al. 2004; Laskowski et al. 1993, 1996; Rieping et al. 2014; Shen and Bax 2007; Vranken and Rieping 2009), and those that consider the NOESY experiments for their structure validation (Huang et al. 2005).

**Fig. 1:**
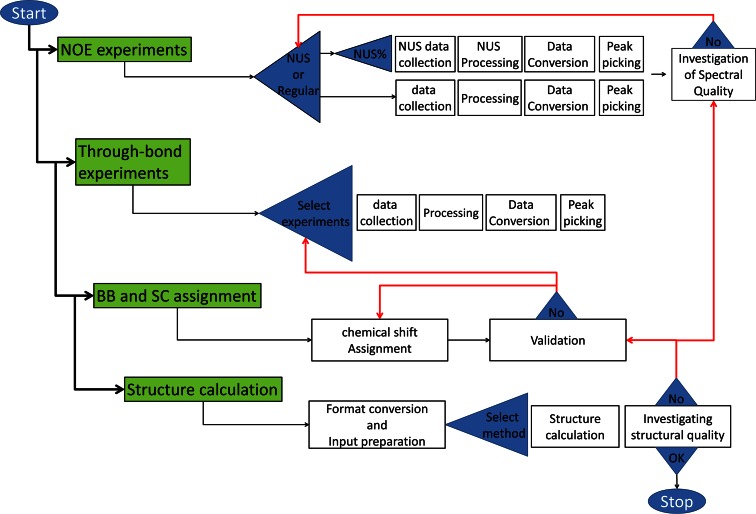
Conventional steps in manual protein structure determination are shown in the *green boxes*. The *blue triangles* indicate decision making steps that user is expected to perform. The *red-lines* show feedback loops from validation steps to the prior steps

The scheme shown in Fig. 1 summarizes various choices and validation steps involved in conventional protein structure determination in the absence of automation. Decisions at the many steps are made according to knowledge and experience and are difficult to document and thus reproduce. User-friendly validation tools are frequently lacking for intermediate steps, and the preparation of input data for structure calculation depends on the program that will be used. If the outcome of the final structure validation is satisfactory, then the process stops. Otherwise, one needs to go back to every step of the process for more precise validation and necessary adjustments.

We introduce here a framework for the process of structure calculation, that a) provides a guideline towards simplifying the process for users with limited NMR background, b) removes the necessary human intervention in data conversion and preparing inputs for discrete steps of the process, c) accelerates the structure calculation process by interconnecting different software packages, d) incorporates validation methods to avoid error accumulation and propagation, and e) incorporates user-friendly refinement modules so the users can perform adjustments whenever needed. Validation is accomplished through statistical analysis and graphical user interfaces that allow results to be compared with underlying data. Smaller and well-behaved proteins are most amenable to full automation, but the framework can be adapted to deal with larger and less well-behaved targets.

## Materials and methods

### Organization

**Fig. 2:**
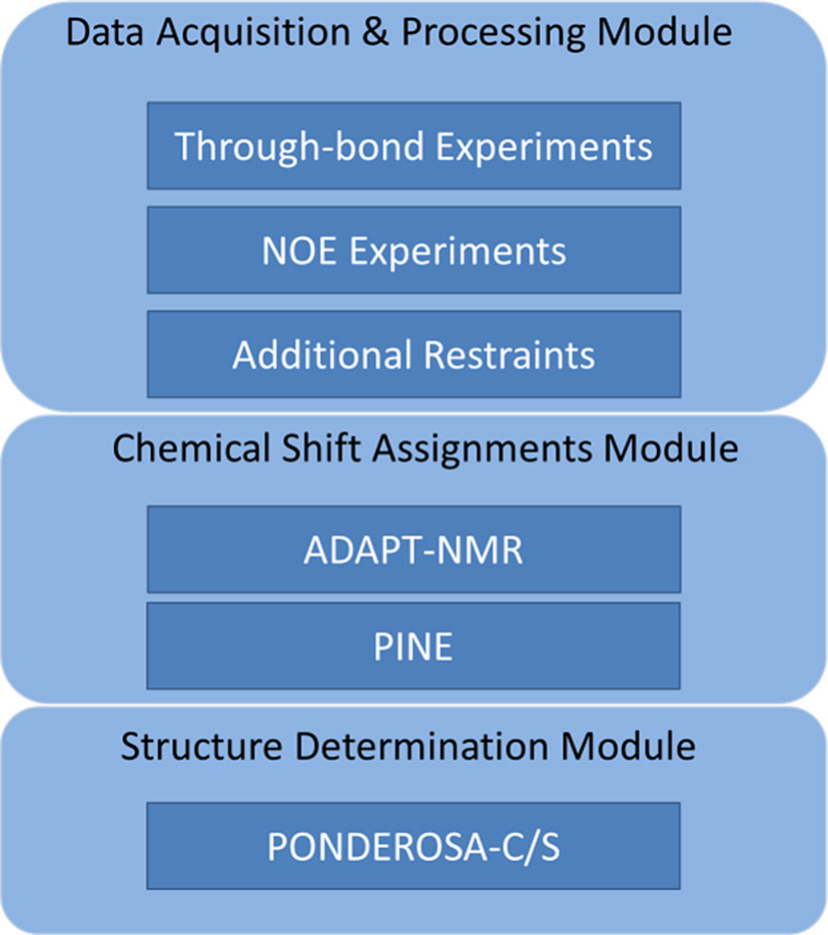
Overall structure of the NMRFAM structure determination framework

Our approach is organized into three steps: (a) data acquisition and processing (including peak picking), (b) chemical shift assignment, and (c) structure determination. NMRFAM-SDF is an object-oriented framework that implements the three steps of this process (Fig. 2), and automatically performs the necessary interconnections between each step. The organization of the modules in this framework is optimized and aimed at complete fully-automated structure determination for well-behaved proteins. After the NMR sample is inserted into the NMR spectrometer, the remaining steps are executed effortlessly leading to structure calculation and refinement. However, for more challenging protein targets, the validation tools identify problems and guide the user to modify the strategy in order to overcome them. The object-oriented organization supports utilities that enable the substitution of every module while maintaining the workflow of the framework. The modules of the framework are described in the following three sections.

### Data acquisition and processing module

**Fig. 3:**
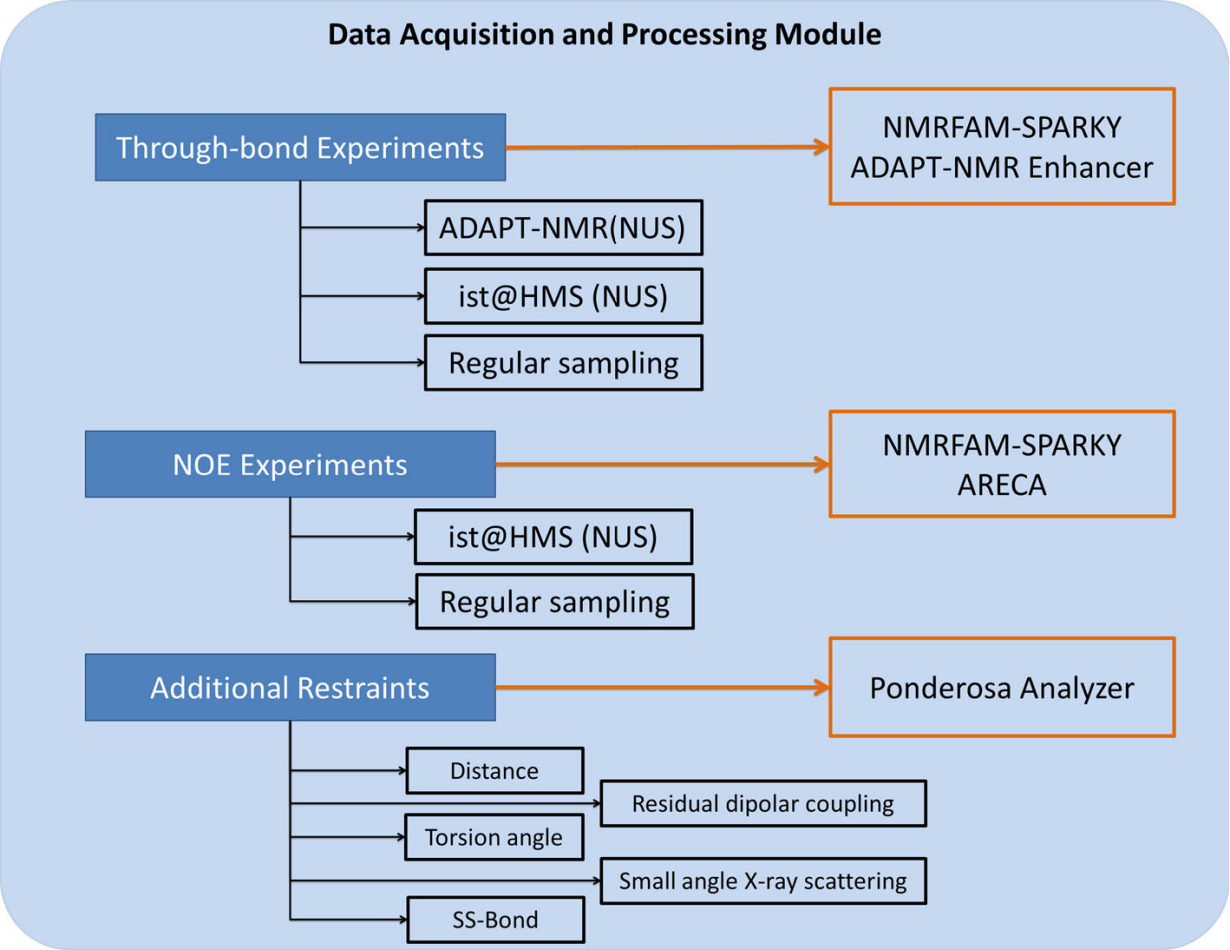
The data acquisition and processing module

The ‘data acquisition and processing’ module consists of three units that focus, respectively, on through-bond experiments, through-space (NOE) experiments, and additional restraints. The tools currently implemented in this module are shown in Fig. 3. Each unit of the module provides a number of options for performing the targeted task (shown as connected boxes in Fig. 3). Orange boxes identify the associated validation tools for each unit.

#### Through-bond experiments

NMRFAM-SDF provides three choices for through-bond experiments: (a) ADAPT-NMR, which uses a non-uniform sampling approach by collecting 3D spectra as tilted 2D planes; (b) non-uniform sampling with iterative soft thresholding (ist@HMS) (Hyberts et al. 2012) with two options for scheduling (default) (Hyberts et al. 2012) or (alternative) NUS-Score (Aoto et al. 2014), and with two options for reconstructing the spectra (default) ist@HMS or (alternative) the much faster NESTA (Sun et al. 2015); and (c) regular sampling by conventional 3D or 4D NMR experiments. Peak picking is an integrated part of ADAPT-NMR, which also achieves probabilistic chemical shift assignments. For the two other options, a peak picking step is required. For these two options, NMRFAM-SDF uses an enhanced approach to the restricted peak picking (Lee et al. 2014b). The validation component, ADAPT-NMR Enhancer, can be used for investigating and validating the results of the tilted-plane data collection and chemical shift assignment. NMRFAM-SPARKY (Lee et al. 2014b) can be used for validating the resolution and sensitivity of spectra collected by options (b) or (c).

#### Through-space (NOE) experiments

NMRFAM-SDF provides two options for collecting NOE experiments: non-uniform sampling with ist@HMS, or regular sampling. Although these options are suitable for well-behaved proteins, the importance of NOESY experiments to achieve proper structural folds makes the validation of through-space experiments crucial. NMRFAM-SPARKY is equipped with tools that map and transfer the chemical shift assignments from the through-bond experiments onto NOESY spectra (two-letter code: ta). The resulting map can be visualized and used to evaluate the quality (resolution and sensitivity) of the spectra. Additionally, our chemical shift validation software, ARECA (Dashti et al. 2015), is used to evaluate the consistency between the assignments and the NOESY spectra (or the corresponding peak lists).

#### Additional restraints

Additional restraints can be incorporated on the basis of the user’s knowledge of the protein under investigation, from manually analyzed experiments (disulfide bonds, residual dipolar coupling, small-angle scattering, or other sources). These additional restraints can be used as auxiliary information to help with the structure determination and/or to validate the final structure.

### Chemical shift assignment module

**Fig. 4:**
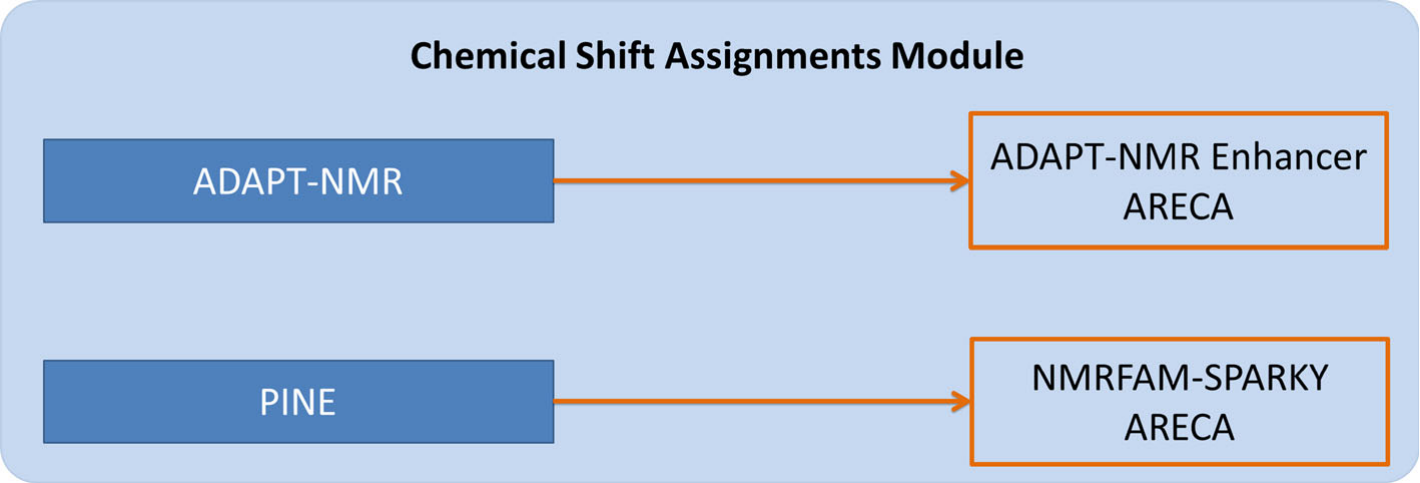
Different computational options in the chemical shift assignment module

The chemical shift assignment module consists of two packages for assigning backbone and side chain atoms. Figure 4 illustrates these packages and their validation tools. When the user selects ADAPT-NMR, assignments are generated automatically during the Bayesian NUS data acquisition. The PINE package facilitates chemical shift assignments from the alternative approaches that generate peak lists associated with particular NMR experiments.

ADAPT-NMR Enhancer and ARECA can be used to validate the chemical shift assignments generated by ADAPT-NMR. Validation of PINE’s output can be performed by PINE-SPARKY (Lee et al. 2009) (incorporated into NMRFAM-SPARKY), or the ARECA package.

### Structure determination module

**Fig. 5:**
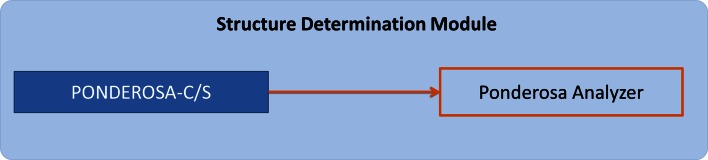
Structure determination module

The core of the structure determination module is the PONDEROSA-C/S package (Fig. 5), which uses the outcomes of the assignments module, the NOE experiments (either raw spectra, refined peak lists, or unrefined peak lists) and the additional restraints for initiating and completing the structure determination step (distance, angle, RDC and SAXS). Cyana (Güntert 2004) formatted files are required for restraints (the Ponderosa Server interconverts these between Cyana and Xplor-NIH formats) with the exception of the raw output from SAXS, which is supported by Xplor-NIH (Kuszewski et al. 2004; 2008; Schwieters et al. 2003). This module is started automatically in our approach unless the user elects to deploy other methods for preparing the input data. Ponderosa Analyzer can be used to validate, evaluate, and adjust the violations in the calculated structure.

## Results

In this section, we discuss applications of NMRFAM-SDF and demonstrate the use of different options within this framework. In all but one case, the proteins chosen for these illustrations are ones with manually determined structures deposited in the PDB, which could be used for comparison; they include targets used in the CASD-NMR competitions. The protein sample conditions are provided in the supplementary materials Table S1.

### [U-^13^C, U-^15^N]-brazzein (53 amino acid residues)

**Fig. 6:**
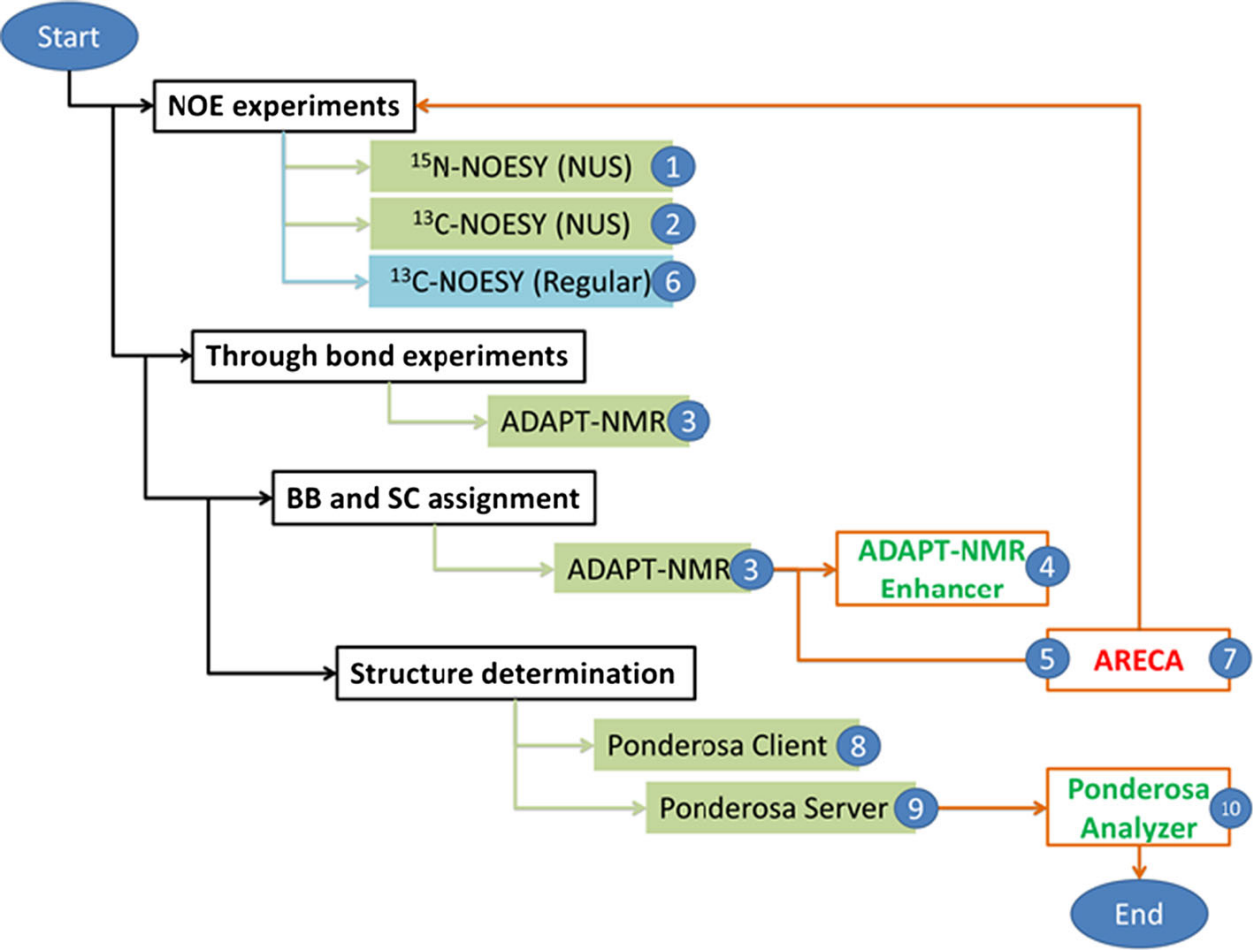
Workflow from the NMRFAM-SDF used for the automated determination of the 3D structure of the protein brazzein. The *black boxes* show different modules of the workflow. The *filled boxes* show the methods used for every module; of these, the *green boxes* indicate steps performed automatically and the *blue box* indicates that ^13^C-NOESY (*regular*) data were collected in response to a validation step. Validation methods are shown within the *orange boxes*. ADAPT-NMR Enhancer and Ponderosa Analyzer confirmed the quality of the data. ARECA reported a high number of suspicious chemical shift assignments at step (5). This prompted the collection of a ^13^C-NOESY spectrum by regular sampling, which resulted in an acceptable ARECA score at step (7). Steps 8–10 resulted in a structure that passed validation

The framework used in this structure determination is shown in Fig. 6.

Steps 1 and 2 (NOESY data collection): Non-uniform sampled data (at a level of 25 %) were collected on a Varian 600 MHz spectrometer; the ist@HMS package was used for scheduling, data collection, and reconstruction of both the ^15^N- and ^13^C-editted NOESY spectra (23 h for each experiment). The Ponderosa Client program was used for peak picking.

**Fig. 7:**
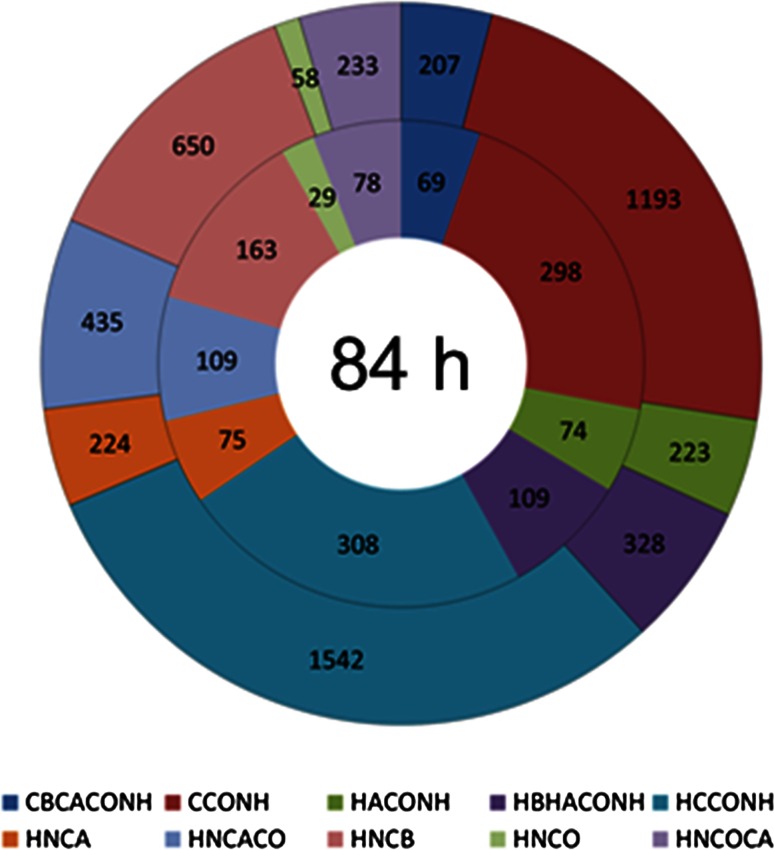
Ten experiments used in ADAPT-NMR. The experiments are color-coded according to the *key at the bottom of the figure*, and the total elapsed time for data collection and chemical shift assignment was 84 h. The *inner layer of the graph* shows the elapsed time for data collection and chemical shift assignment of every tilted plane (in minutes), and the *outer layer* shows the total time for every experiment (in minutes)

Step 3 (through-bond data collection and assignment): ADAPT-NMR was used for data collection and assignment of the backbone and side chain atoms. Figure 7 shows the collected experiments and elapsed time for both data acquisition and chemical shift assignments.

Step 4 (validation with ADAPT-NMR Enhancer): ADAPT-NMR Enhancer was utilized to validate the chemical shift assignments by checking them against the spectral data.

**Fig. 8:**
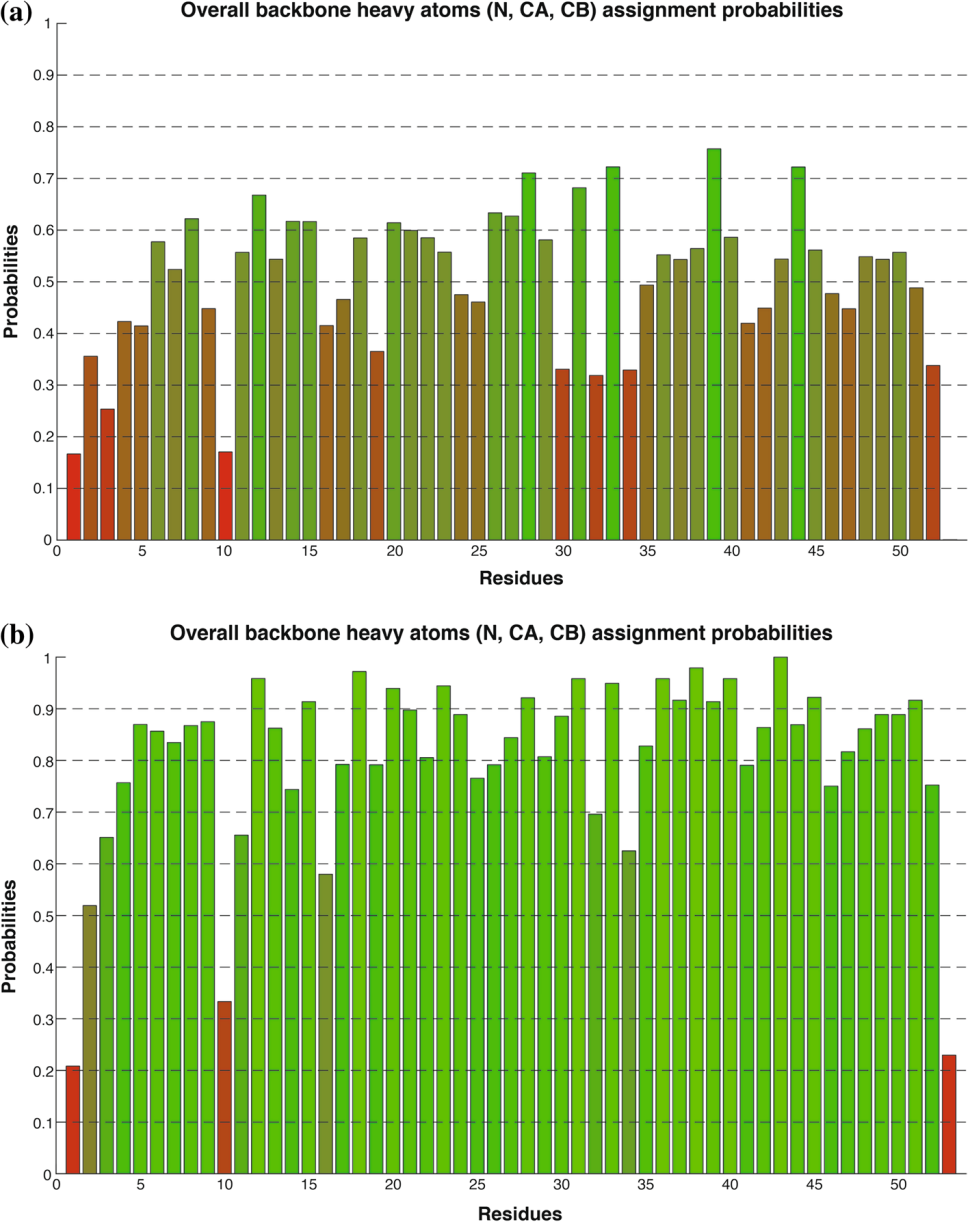
ARECA’s overall probabilities for the backbone (plus CB) heavy atoms of the brazzein protein. **a** ARECA output from the non-uniformly sampled ^15^N- and ^13^C-edited NOESY spectra. **b** ARECA’s output from the non-uniformly sampled ^15^N-edited and regularly-sampled ^13^C-edited NOESY spectra. In these plots, the residues are shown on the *x*-axis and the *y*-axis indicates the overall probabilities of the heavy atoms. In ARECA, probabilities lower than 50 % (indicated by *red bars*) indicate possible problematic assignments

Step 5 (validation with ARECA): The ARECA package was used to evaluate the consistency between the NOESY spectra and the assignments. ARECA flagged 133 atoms (25.3 % of the total number of assigned atoms) with low probabilities (probabilities less than 50 % are considered low). Because more than 5 % of the atoms were flagged, inconsistency between the assignments and the NOESY spectra was considered a possibility. Figure 8a shows ARECA’s report on the overall probabilities of the backbone heavy atoms.

Step 6 (NOESY data collection): Because ARECA’s report on the NOESY data was unsatisfactory, the NOESY spectra were inspected manually with NMRFAM-SPARKY, and a regularly-sampled ^13^C-edited NOESY spectrum was collected, and used to replace the ^13^C-NOESY (NUS) data.

Step 7 (validation with ARECA): The regularly-sampled ^13^C-edited NOESY spectrum, along with the non-uniformly sampled ^15^N-edited NOESY spectrum, were used to recalculate ARECA’s probabilities. ARECA flagged only 13 atoms (2.48 %) with low probabilities, which was a significant improvement on the consistency between the new set of NOESY spectra and the assignments. Figure 8b shows the overall probabilities of the backbone heavy atoms as reported by ARECA.

Step 8 (Structure calculation with PONDEROSA-C/S): Ponderosa Client submitted the complete validated data package to the Ponderosa Server. The refinement option was set to use Cyana for NOE assignment and structure calculation, and Xplor-NIH for water refinement (PONDEROSA refinement option).

**Fig. 9:**
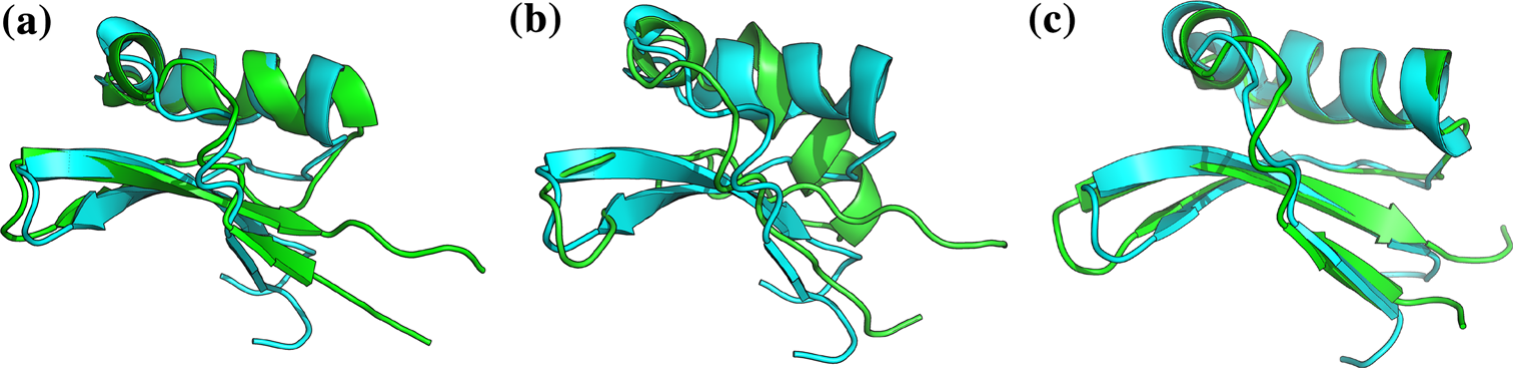
Structures of brazzein protein with achieved automatically with NMRFAM-SDF (*green*) superimposed on the manually refined structure (*cyan*). **a** Structure generated with the non-uniformly sampled ^15^N-edited NOESY spectrum and the regularly-sampled ^13^C-edited NOESY spectrum. **b** Structure generated with the non-uniformly sampled ^15^N- and ^13^C-edited NOESY spectra. **c** Structure generated with the regularly-sampled ^15^N- and ^13^C-edited NOESY spectra and manual chemical shift assignments

Step 9 (Structure evaluation with Ponderosa Analyzer): Table S2(a) shows the PONDEROSA-C/S and PSVS (Bhattacharya et al. 2006) structure validation reports for this structure. These reports on the quality of the structure were satisfactory; therefore, the structure determination was considered to be successful, and the process was stopped. To further evaluate the results of this workflow, the chemical shift assignments and the calculated structure were compared with the manually derived assignments (BMRB entry 16215) and structure of the protein (PDB entry 2LY5) (Cornilescu et al. 2013). Comparison of chemical shifts assignments indicated that 84.3 % of the overall backbone and side chain assignments achieved automatically were in agreement with those deposited in BMRB. We consider the deposited assignments to be correct, because they were obtained in the course of structure determination and refinement. Despite the 15.7 % erroneous assignments, the structure calculated automatically contained the expected strands and helices and had a backbone RMSD of 1.67 Å to the manually refined structure (Fig. 9a).

In order to test whether the early validation step was necessary for achieving a good structure, we used the non-uniformly sampled ^15^N- and ^13^C-edited NOESY spectra as input to the Ponderosa Server (despite the 25.3 % assignments flagged by ARECA). The resulting structure (Fig. 9b) was missing the three strands and had a backbone RMSD of 2.91Å to the manually determined structure. Table S2(b) shows the structure validation reports for this structure generated by PONDEROSA-C/S and PSVS.

To evaluate the influence of erroneous assignments on the quality of the structure, we used the regularly-sampled ^15^N- and ^13^C-edited NOESY spectra and correct manual assignments (BMRB entry 16215) as input to NMRFAM-SDF. The resulting structure (Fig. 9c) had a backbone RMSD of 1.22 Å from the manually refined structure (PDB entry 2LY5). From the validation report (Table S2(c)), it is clear that the overall quality of the structure is improved. However, the original structure determined with minimal human intervention (Fig. 9a) was of sufficient quality that it could have been used as a starting point for manual validation and refinement of the structure.

### [U-^13^C, U-^15^N]- chlorella-ubiquitin (76 amino acid residues)

**Fig. 10:**
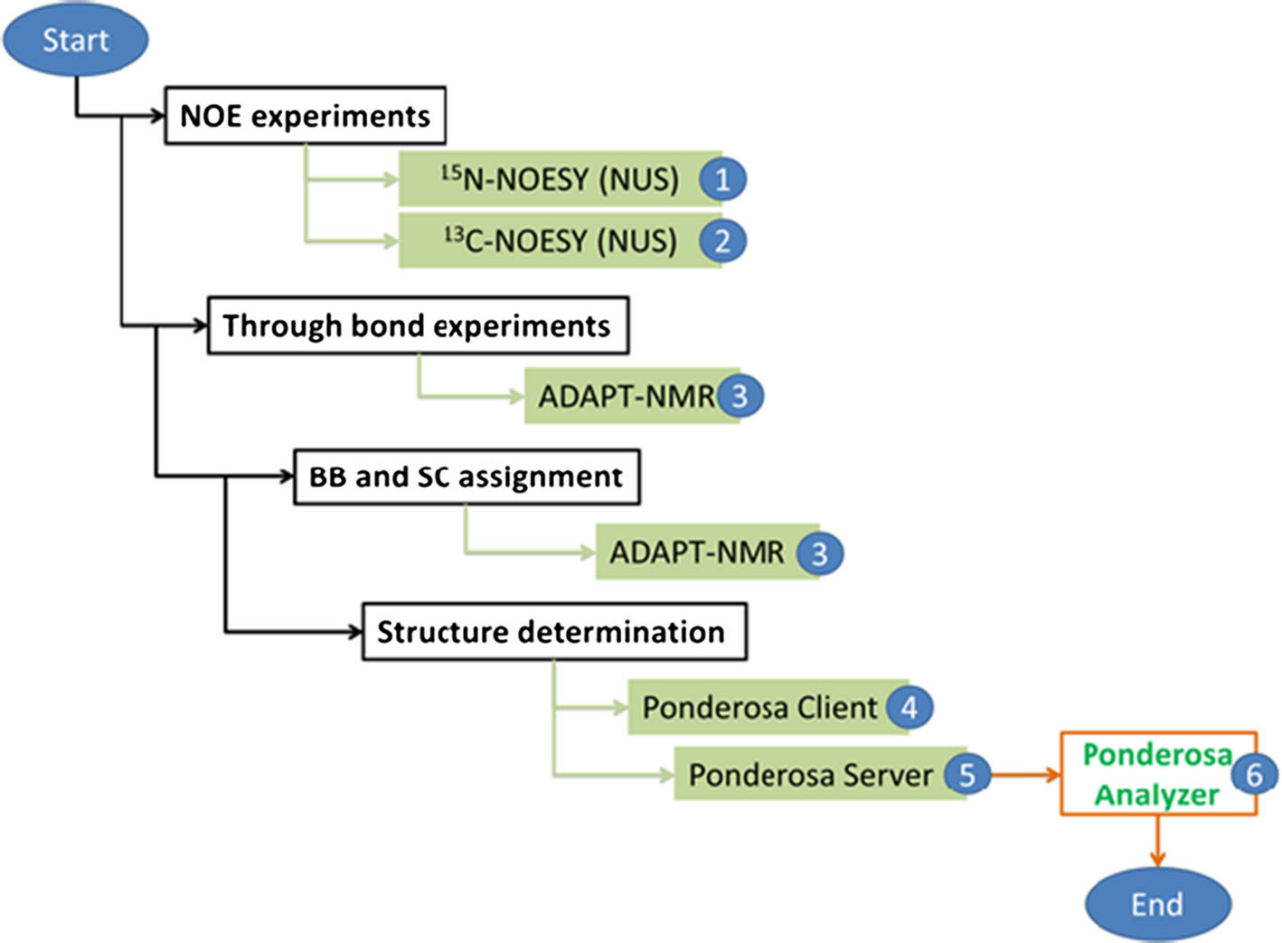
NMRFAM-SDF workflow used in the fully automated structure determination of ubiquitin

A fully automated workflow (Fig. 10) was used for this protein, which was prepared by cell-free protein production.

Steps 1 and 2 (NOESY data collection): ^15^N- and ^13^C-edited NOESY spectra were recorded on a Varian 800 MHz spectrometer equipped with cryogenic probe and processed using the ist@HMS package. The ^13^C-NOESY data were collected at a sampling level of 64 % (42 h), and the ^15^N-NOESY data were collected at a sampling level of 36 % (24 h).

**Fig. 11:**
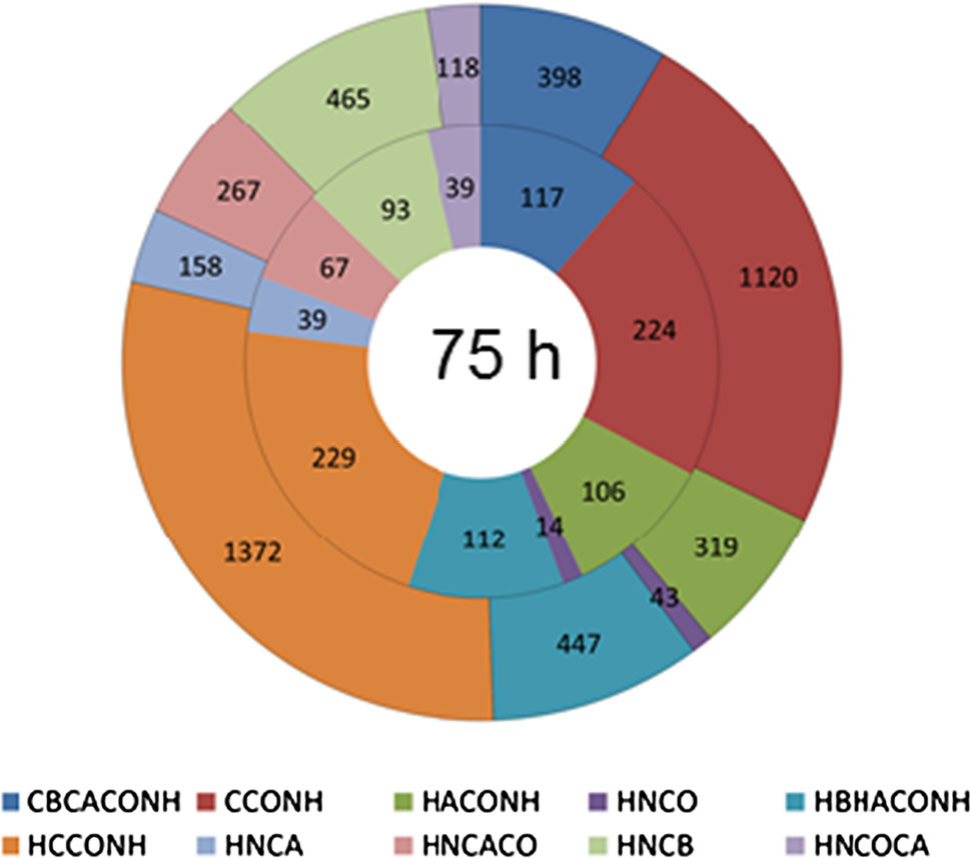
The total time for data collection and chemical shift assignments by ADAPT-NMR was 75 h. For every experiment (*color-coded* according to the* key at the bottom of the figure*), the *inner layer* shows the elapsed time for data collection and chemical shift assignment of a tilted plane (in minutes) and the *outer layer* shows the total elapsed time for the experiment (in minutes)

Step 3 (through-bond data collection and assignment): Non-uniform sampling with ADAPT-NMR was used for data collection (Fig. 11) and assignments of the backbone and side chain atoms.

Step 4 and 5 (Structure calculation with PONDEROSA-C/S): The Ponderosa Client was used for peak picking of the NOESY spectra, and for submitting the job to the Ponderosa Server with the PONDEROSA refinement option.

**Fig. 12:**
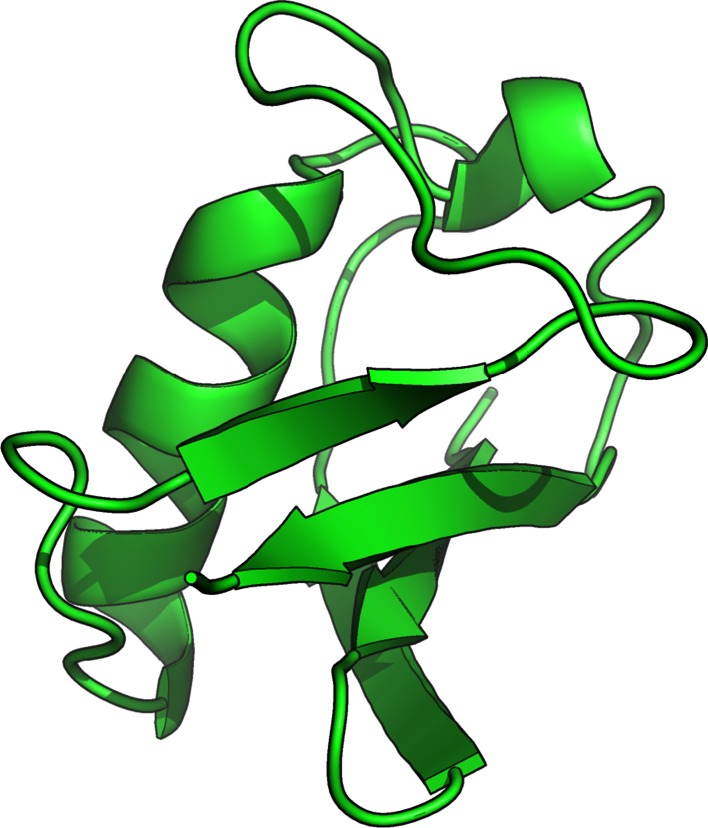
Structure of chlorella-ubiquitin obtained by using the NMRFAM-SDF

Step 6 (Structure evaluation with Ponderosa Analyzer): The structure validation reports generated by PONDEROSA-C/S and PSVS are shown in Table S3. On the basis of the validation statistics, the structure was considered acceptable, and the process was stopped. Because the coordinates of the manually determined structure were not reported ((Ikeya et al. 2009) and BMRB entry 16228), we show only the structure calculated by using the NMRFAM-SDF (Fig. 12).

**Fig. 13:**
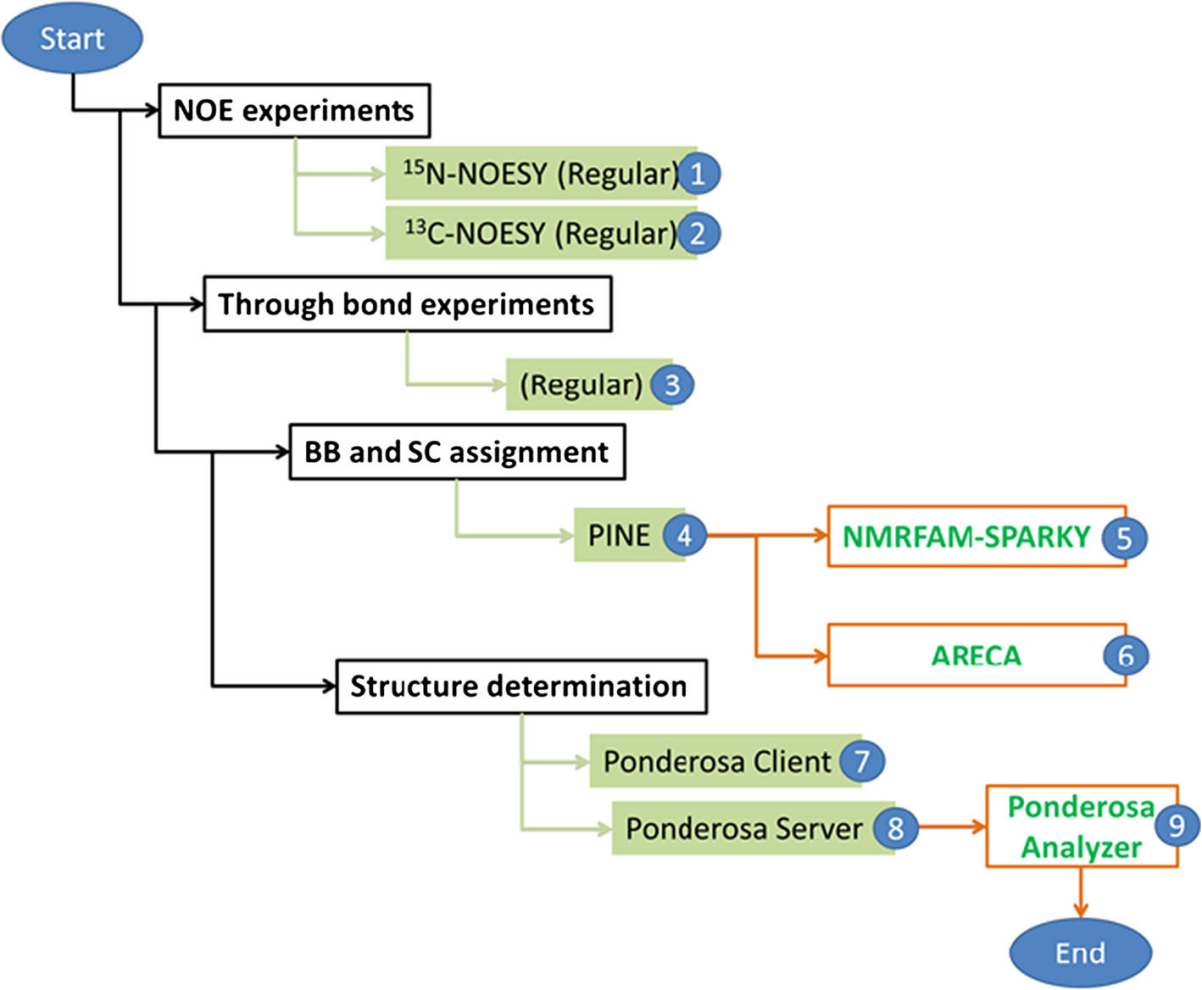
NMRFAM-SDF workflow for data collected conventionally

The two examples shown above used ADAPT-NMR for non-uniform data collection and assignments. In the following two examples, we consider a process in which through-bond experiments are collected manually, peak picking is performed with NMRFAM-SPARKY, and the PINE package is used for chemical shift assignments. The NMRFAM-SDF for this protocol (Fig. 13) was used to calculate the 3D structures of human ubiquitin and IscU (D39A).

### [U-^13^C, U-^15^N]-human ubiquitin (76 amino acid residues)

Steps 1 and 2 (NOESY data collection): ^15^N- and ^13^C-edited NOESY spectra were collected with regularly-sampled time schedules.

Step 3 (through-bond data collection): Data from through-bond experiments were collected with regularly-sampled time schedules for eight experiments (2D ^1^H-^15^N-HSQC, 2D ^1^H-^13^C-HSQC, 3D CBCA(CO)NH, 3D C(CO)NH, 3D HBHA(CO)NH, 3D HCCH-TOCSY, 3D H(CCO)NH, and 3D HNCACB). NMRFAM-SPARKY was used to prepare peak lists from these experiments.

Step 4 (chemical shift assignment): These peak lists were used for chemical shift assignment with the PINE package.

Step 5 (validation with NMRFAM-SPARKY): The first step of validation was to use PINE-Sparky to evaluate the assignments. For this protein, the chemical shift assignments of 55 atoms out of 760 (7 %) were manually modified during this validation process.

Step 6 (Validation with ARECA): The ARECA package was used to validate the assignments against NOESY spectra. ARECA reported 21 atoms (2.7 %) with low probabilities, which is considered within the acceptable range (fewer than 5 % of the total number of assigned atoms); therefore, no further data collection was needed.

Step 7 (Structure calculation with Ponderosa): Ponderosa Client was used for peak picking of the NOESY spectra and for submitting the job to PONDEROSA-C/S with the “PONDEROSA refinement option”.

**Fig. 14:**
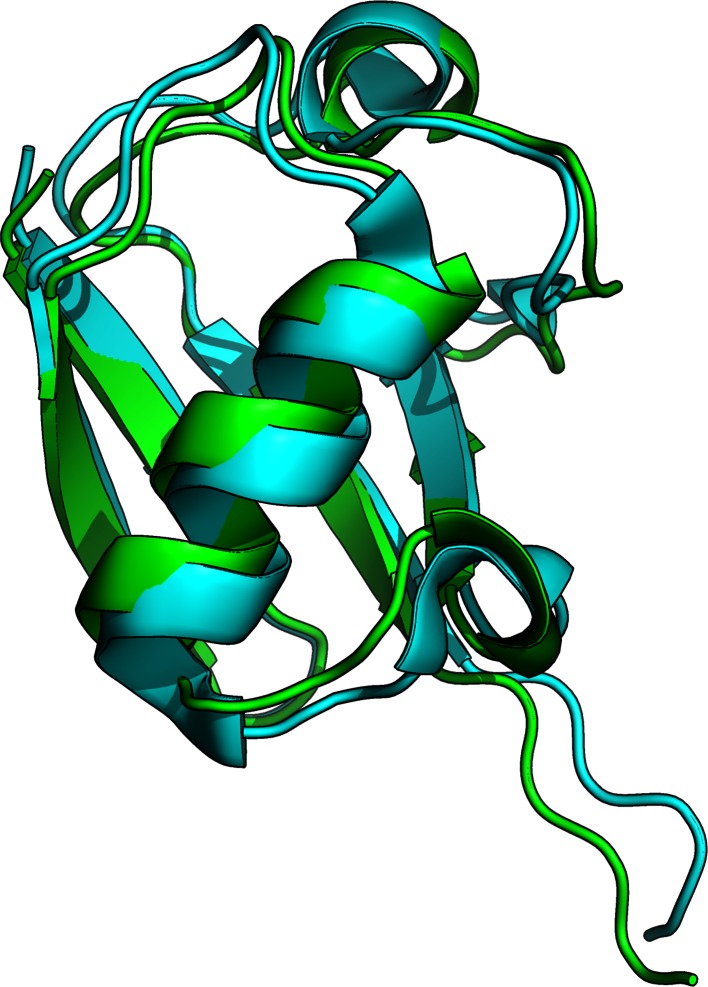
Superimposition of the manual structure (*cyan*) and automated structure (*green*) of human ubiquitin

Step 8 (Structure evaluation with Ponderosa Analyzer): Table S4 shows validation reports for the structure generated by PODEROSA-C/S, which were considered satisfactory. For further evaluation of the structure, we compared the structure determined with this workflow against the manually-refined structure (PDB entry 1D3Z). The backbone RMSD between the two structures was 0.99 Å (Fig. 14), which indicates close match between the determined structures and shows accuracy of the framework.

### [U-^13^C, U-^15^N]-IscU (D39A) (128 amino acid residues)

**Fig. 15:**
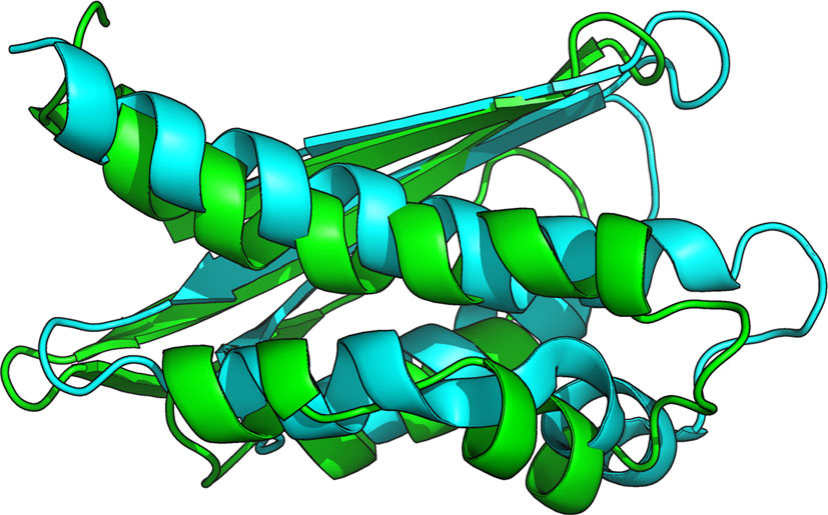
Superimposition of the manual structure (*cyan*) and automated structure (*green*) of the protein IscU (D39A)

The structured variant (D38A) of the protein IscU from *Escherichia coli* (IscU (D39A)) was considered as another example for this alternative workflow (Fig. 13). Because of dynamics of the protein in solution (Kim et al. 2012), residual dipolar coupling (RDC) data were used as “Additional Restraints” in the framework. The Ponderosa Client was used for peak picking the NOESY spectra and submitting a job to the Ponderosa Server. Table S5 shows the PONDEROSA-C/S and the PSVS outputs for the structure generated by the workflow. In addition to the acceptable structure validation statistics, comparison between the ordered regions (residues 19-60, 68-125) of the manually derived structure (Kim et al. 2012) (PDB entry 2KQK, BMRB entry 16603) and the structure calculated by NMRFAM-SDF resulted in a backbone RMSD of 0.99 Å (Fig. 15).

### [U-^13^C, U-^15^N]-HR6470A (69 amino acid residues)

**Fig. 16:**
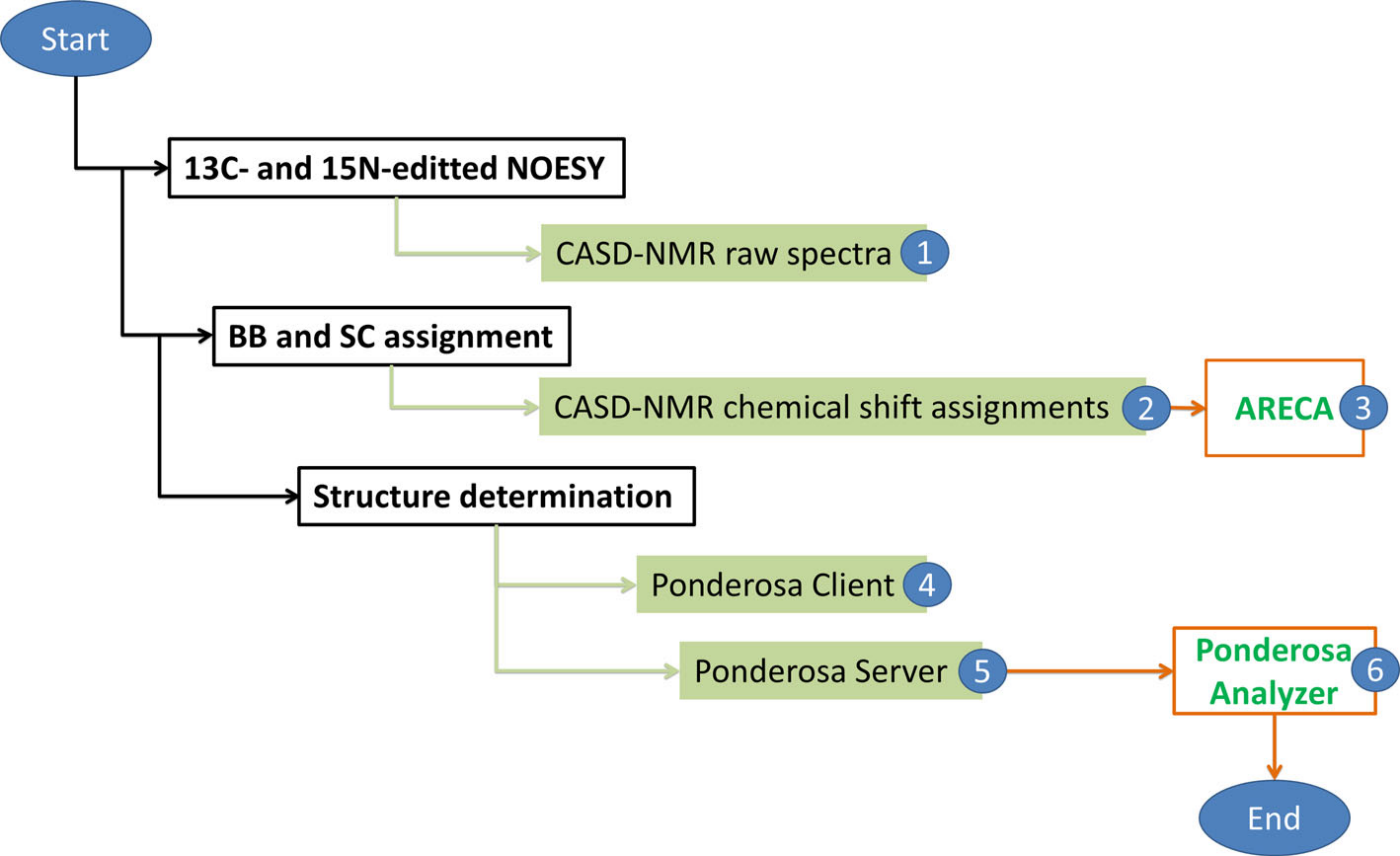
NMRFAM-SDF workflow for CASD-NMR target protein HR6470A. In this workflow, ARECA was used to validate the chemical shift assignments, Ponderosa Client was used for peak picking of the NOESY spectra and also submitting the input files to the Ponderosa Server for structure calculations, and Ponderosa Analyzer was used to validate the structure

In this final example, which involves the second round CASD-NMR target protein HR6470A, the input data to the framework were the raw ^13^C- and ^15^N-filtered NOESY spectra and the chemical shift assignments provided for the competition. The NMRFAM-SDF workflow for this example is shown in Fig. 16.

Steps 1 and 2 (Peak lists and assignments): The raw ^13^C- and ^15^N-filtered NOESY spectra and the chemical shift assignments of protein HR6470A were used as the inputs to the framework. Ponderosa Client was used to peak-pick the spectra.

Step 3 (Validation with ARECA): The ARECA package was used to validate the assignments against the NOESY peak lists. ARECA reported only 6 assignments (0.70 %) with low probability, which is considered within the acceptable range (fewer than 5 % of the total number of assigned atoms); therefore, the quality of the chemical shifts assignments was considered to be satisfactory.

Step 4 (Structure calculation with Ponderosa): Ponderosa Client was used to prepare input submitted to PONDEROSA-C/S with the “PONDEROSA refinement option”.

**Fig. 17:**
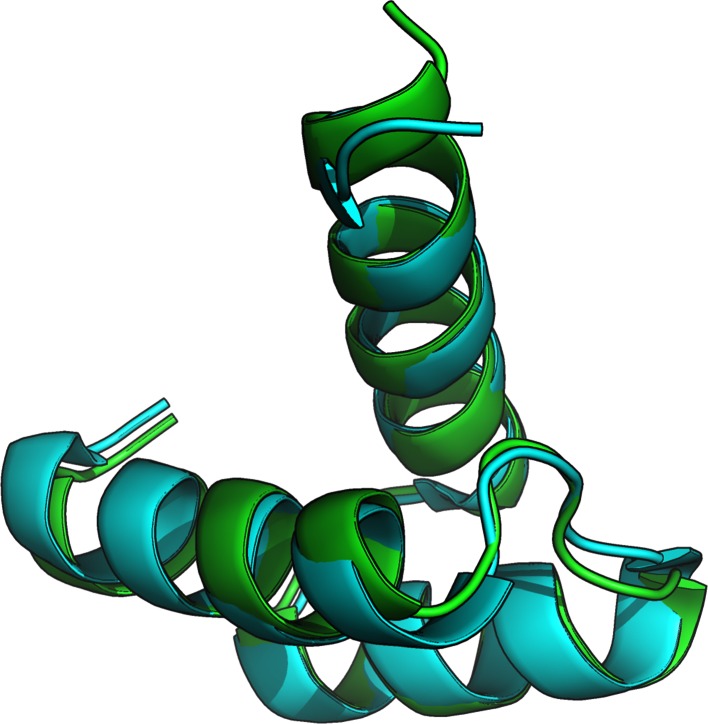
Superposition of the structure of protein HR6470A calculated by NMRFAM-SDF (*green*) with the manually determined structure (*cyan*) deposited as PDB entry 2L9R

Step 5 (Structure evaluation with Ponderosa Analyzer): The statistics for structure validation generated with the Ponderosa Analyzer indicated satisfactory results (Table S6); thus the structure was deemed to be acceptable. Comparison of this structure with the manually determined structure (PDB entry 2L9R) resulted in a backbone RMSD of 0.51 Å (Fig. 17).

## Conclusions

The process of protein structure determination by NMR spectroscopy consists of several computationally demanding steps. In order to develop high-throughput methods and to simplify the process into a robust approach for use by non-experts, algorithms for automation of discrete steps have been introduced. To accomplish this goal, the need for a user-friendly approach that includes several practical validation steps is inevitable. We have introduced a framework for the process of protein structure determination (NMRFAM-SDF) that is designed to achieve four goals: (a) to accelerate the structure determination process by removing human intervention, (b) to provide a workflow for fully automated structure determination for well-behaved proteins, (c) to provide unbiased validation tools for every step of the process, (d) to provide user-friendly refinement tools to prevent error propagation in the process. We have shown here that these steps can be assembled into various workflows and used to solve structures of relatively small test proteins labeled uniformly with ^13^C and ^15^N. The applicability of this approach to the broader landscape of structure determination remains to be tested thoroughly, although we and others have shown success in using components of the framework, such as PINE and PONDEROSA-C/S, with much larger proteins. Semi-automated inspection and validation tools will be particularly useful for more complex proteins. Additional validation tools are planned, and NMRFAM-SDF will provide a solid foundation for these extensions.

## Electronic supplementary material

Supplementary material 1 (DOCX 34 kb)
