# Supplementary material for: NMRFAM-SDF: a protein structure determination framework
Source: J Biomol NMR. 2015 Apr 22;62(4):481–95. doi: 10.1007/s10858-015-9933-8 (PMC4569665; doi:10.1007/s10858-015-9933-8)
Supplement: Supplementary file 1 — Supplementary material 1 (DOCX 34 kb) [file 10858_2015_9933_MOESM1_ESM.docx]

**NMRFAM-SDF: a protein structure determination framework**

Hesam Dashti • Woonghee Lee • Marco Tonelli • Claudia C. Cornilescu • Gabriel Cornilescu •

Fariba M. Assadi-Porter • William M. Westler • Hamid R. Eghbalnia • John L. Markley

**Supplementary material**

| **Table S1.** Sample conditions of the [U-^13^C, U-^15^N]-proteins studied. |
| --- |
| \|  \| Temperature \| pH \| Protein concentration \| Buffer composition \| \| --- \| --- \| --- \| --- \| --- \| \| brazzein \| 310 \| 5.2 \| 1.5 mM \| 10 mM NaCl, 90%/10% H_2_O/D_2_O \| \| chlorella ubiquitin \| 298 \| 6.6 \| 1.1 mM \| 10 mM phosphate, 0.04% NaN_3_, 90%/10% H_2_O/D_2_O \| \| human ubiquitin \| 308 \| 6.5 \| 0.7 mM \| 10 mM phosphate, 93%/7% H_2_O/D_2_O \| \| IscU(D39A) \| 298 \| 8 \| 2.0 mM \| 20 mM TRIS,  5 mM dithiothreitol,  150 mM NaCl, 0.5 mM EDTA, 0.02 % NaN_3_, 93%/7% H_2_O/D_2_O \| \| HR6470A \| 298 \| 6.5 \| 0.69 mM \| 90%/10% H_2_O/D_2_O \| |

|  |
| --- |
| \| **Table S2.** Statistics from the validation reports for the NMR structures of brazzein determined under NMRFAM-SDF by PONDEROSA-C/S. (a) Structure generated with the non-uniformly sampled ^15^N-edited NOESY spectrum and the regularly-sampled ^13^C-edited NOESY spectrum. (b) Structure generated with the non-uniformly sampled ^15^N- and ^13^C-edited NOESY spectra. (c) Structure generated with the regularly-sampled ^15^N- and ^13^C-edited NOESY spectra and manual chemical shift assignments. \| \| \|  \| \| --- \| --- \| --- \| --- \| \|  \| (a) \| (b) \| (c) \| \| Conformationally restricting distance constraints \|  \|  \|  \| \| Short Range [(i– j) <= 1] \| 443 \| 129 \| 567 \| \| Medium Range [1 < (i – j) ≤ 5] \| 177 \| 20 \| 158 \| \| Long Range [(i – j) > 5] \| 243 \| 19 \| 295 \| \| Total \| 863 \| 168 \| 1020 \| \| Dihedral angle constraints \|  \|  \|  \| \| ϕ \| 40 \| 47 \| 43 \| \| ψ \| 43 \| 48 \| 41 \| \| Hydrogen-bond constraints \| 0 \| 0 \| 0 \| \| CYANA target function [Å] \| 0.43 \| 0.01 \| 0.43 \| \| Average rmsd to the mean CYANA coordinates [Å] \|  \|  \|  \| \| Backbone heavy atoms N, Cα, C′ (2–52) \| 0.1 \| 1.3 \| 0.1 \| \| All heavy atoms (2–52) \| 0.7 \| 2.0 \| 0.5 \| \| PROCHECK Z-scores (φ and Ψ/all dihedral angles ) \| -3.11/-6.56 \| -2.20/-5.03 \| -2.87/-5.74 \| \| MOLPROBITY Mean score/clash score \| -2.63/24.23 \| -0.15/9.77 \| -0.56/12.13 \| \| Ramachandran plot summary ordered residue ranges [%] \|  \|  \|  \| \| Most favored regions \| 90.2 \| 96.3 \| 86 \| \| Allowed regions \| 7.8 \| 3.7 \| 13.9 \| \| Disallowed regions \| 2.0 \| 0.0 \| 0.1 \| \| Average number of distance constraint violations per CYANA conformer \|  \|  \|  \| \| 0.2 – 0.5 Å \| 0 \| 0 \| 1 \| \| > 0.5 Å \| 0 \| 0 \| 0 \| \| Average number of angle constraint violations per CYANA conformer \|  \|  \|  \| \| > 10° \| 0 \| 0 \| 0 \| |

|  |
| --- |
| \| **Table S3.** Statistics for the NMR structure of chlorella-ubiquitin determined under NMRFAM-SDF by PONDEROSA-C/S. \| \| \| --- \| --- \| \| Conformationally restricting distance constraints \|  \| \| Short Range [(i– j) <= 1] \| 706 \| \| Medium Range [1 < (i – j) ≤ 5] \| 213 \| \| Long Range [(i – j) > 5] \| 333 \| \| Total \| 1252 \| \| Dihedral angle constraints \|  \| \| ϕ \| 66 \| \| ψ \| 67 \| \| Hydrogen-bond constraints \| 4 \| \| CYANA target function [Å]  Average rmsd to the mean CYANA coordinates [Å] \| 0.67 \| \| Backbone heavy atoms N, Cα, C′ (2–72) \| 0.4 \| \| All heavy atoms (2–72) \| 0.9 \| \| PROCHECK Z-scores (φ and Ψ/all dihedral angles ) \| -2.24/-4.67 \| \| MOLPROBITY Mean score/clash score \| -4.55/35.43 \| \| Ramachandran plot summary ordered residue ranges [%] \|  \| \| Most favored regions \| 95.6 \| \| Allowed regions \| 4.4 \| \| Disallowed regions \| 0 \| \| Average number of distance constraint violations per CYANA conformer \|  \| \| 0.2 – 0.5 Å \| 0 \| \| > 0.5 Å \| 0 \| \| Average number of angle constraint violations per CYANA conformer \|  \| \| > 10° \| 0 \| |

|  |
| --- |
| \| **Table S4.** Statistics for the NMR structure of human ubiquitin determined under NMRFAM-SDF by PONDEROSA-C/S. \| \| \| --- \| --- \| \| Conformationally restricting distance constraints \|  \| \| Short Range [(i– j) <= 1] \| 813 \| \| Medium Range [1 < (i – j) ≤ 5] \| 323 \| \| Long Range [(i – j) > 5] \| 404 \| \| Total \| 1540 \| \| Dihedral angle constraints \|  \| \| ϕ \| 67 \| \| ψ \| 70 \| \| Hydrogen-bond constraints \| 0 \| \| CYANA target function [Å] \| 1.01 \| \| Average rmsd to the mean CYANA coordinates [Å] \|  \| \| Backbone heavy atoms N, Cα, C′ (1-73) \| 0.2 \| \| All heavy atoms (1-73) \| 0.6 \| \| PROCHECK Z-scores (φ and Ψ/all dihedral angles ) \| -1.18/-3.37 \| \| MOLPROBITY Mean score/clash score \| -4.77/36.66 \| \| Ramachandran plot summary ordered residue ranges [%] \|  \| \| Most favored regions \| 99.4 \| \| Allowed regions \| 0.6 \| \| Disallowed regions \| 0.0 \| \| Average number of distance constraint violations per CYANA conformer \|  \| \| 0.2 – 0.5 Å \| 3 \| \| > 0.5 Å \| 0 \| \| Average number of angle constraint violations per CYANA conformer \|  \| \| > 10° \| 0 \| |

|  |
| --- |
| \| **Table S5.** Statistics for the NMR structure of IscU (D39A) determined under NMRFAM-SDF by PONDEROSA-C/S. \| \| \| --- \| --- \| \| Conformationally restricting distance constraints \|  \| \| Short Range [(i– j) <= 1] \| 614 \| \| Medium Range [1 < (i – j) ≤ 5] \| 241 \| \| Long Range [(i – j) > 5] \| 249 \| \| Total \| 1104 \| \| Dihedral angle constraints \|  \| \| ϕ \| 101 \| \| ψ \| 104 \| \| Hydrogen-bond constraints \| 0 \| \| CYANA target function [Å] \| 1.60 \| \| Average rmsd to the mean CYANA coordinates [Å] \|  \| \| Backbone heavy atoms N, Cα, C′ (19-60,68-125) \| 0.7 \| \| All heavy atoms (19-60,68-125) \| 1.2 \| \| PROCHECK Z-scores (φ and Ψ/all dihedral angles ) \| -0.90/-3.78 \| \| MOLPROBITY Mean score/clash score \| -1.91/20.00 \| \| Ramachandran plot summary ordered residue ranges [%] \|  \| \| Most favored regions \| 97.9 \| \| Allowed regions \| 2.1 \| \| Disallowed regions \| 0.0 \| \| Average number of distance constraint violations per CYANA conformer \|  \| \| 0.2 – 0.5 Å \| 0 \| \| > 0.5 Å \| 1 \| \| Average number of angle constraint violations per CYANA conformer \|  \| \| > 10° \| 0 \| |

|  |
| --- |
| \| **Table S6.** Statistics for the NMR structure of CASD-NMR target HR6470A under NMRFAM-SDF by PONDEROSA-C/S. \| \| \| --- \| --- \| \| Conformationally restricting distance constraints \|  \| \| Short Range [(i– j) <= 1] \| 640 \| \| Medium Range [1 < (i – j) ≤ 5] \| 323 \| \| Long Range [(i – j) > 5] \| 226 \| \| Total \| 1189 \| \| Dihedral angle constraints \|  \| \| ϕ \| 45 \| \| ψ \| 46 \| \| Hydrogen-bond constraints \| 0 \| \| CYANA target function [Å] \| 0.64 \| \| Average rmsd to the mean CYANA coordinates [Å] \|  \| \| Backbone heavy atoms N, Cα, C′ (11–60) \| 0.3 \| \| All heavy atoms (11–60) \| 0.8 \| \| PROCHECK Z-scores (φ and Ψ/all dihedral angles ) \| 0.43/-1.01 \| \| MOLPROBITY Mean score/clash score \| -0.36/10.99 \| \| Ramachandran plot summary ordered residue ranges [%] \|  \| \| Most favored regions \| 95.6 \| \| Allowed regions \| 4.4 \| \| Disallowed regions \| 0 \| \| Average number of distance constraint violations per CYANA conformer \|  \| \| 0.2 – 0.5 Å \| 2 \| \| > 0.5 Å \| 0 \| \| Average number of angle constraint violations per CYANA conformer \|  \| \| > 10° \| 0 \| |
